# The Annual of Scientific Discovery; or, Year Book of Facts in Science and Art—Exhibiting the Most Important Discoveries and Improvements in Mechanics, Useful Arts, Natural Philosophy, Chemistry, Astronomy, Meteorology, Zoology, Botany, Mineralogy, Geology, Geography, Antiquities

**Published:** 1850-04

**Authors:** 


Art. VIII.—*The Annual of Scientific Discovery; or Year Book of Facts in Science and Art*—Exhibiting the most important discoveries and improvements in mechanics, useful arts, natural philosophy, chemistry, astronomy, meteorology, zoology, botany, mineralogy, geology, geography, antiquities. Together with a list of recent scientific publications; a classified list of patents; obituaries of eminent scientific men; an index of important papers in scientific journals, reports, etc. Edited by David Wells, of the Lawrence Scientific School, Cambridge, and George Bliss, Jr.

We regret that this valuable work was not received in time for an extended notice in this number. It is a repository of knowledge, of the most useful character, and should command a very extensive patronage. Since we have seen it, we wonder that such a publication should not have been commenced years ago.

We have only space, now, sufficient to announce the publication of this valuable work, and to say that it presents the best dollar's worth of reading that we know of.

The work consists of 392 pages, and the publishers, Gould, Kendall and Lincoln, of Boston, proffer to send it, free of postage, to any one who remits them one dollar.

